# Exogenous Brassinosteroid Promotes Vegetative Growth in the World’s Smallest Water Lily (*Nymphaea thermarum*)

**DOI:** 10.3390/plants15203205

**Published:** 2026-10-19

**Authors:** Bao-Yu Li, Jin-Sheng Xie, Yan-Jie Wang, Qi-Jiang Jin, Dong Xiao, Zhi-Juan Yang, Ying-Chun Xu

**Affiliations:** 1 Sanya Institute of Nanjing Agricultural University, College of Horticulture, Nanjing Agricultural University, Nanjing 210095, China; 2 School of Breeding and Multiplication (Sanya Institute of Breeding and Multiplication), Hainan University, Sanya 572025, China

**Keywords:** *Nymphaea thermarum*, brassinosteroid, dwarfism, transcriptome, cell wall biosynthesis

## Abstract

*Nymphaea thermarum*, the world’s smallest water lily, provides a useful system for investigating hormonal regulation of vegetative growth. This study investigated vegetative growth, hormone immunoreactive estimates, and tissue-specific transcriptional responses to exogenous 24-epibrassinolide (EBL) in *N. thermarum*. Plants were subjected to a concentration gradient of 24-epibrassinolide (EBL; 0, 200, 400, 600, 800, and 1000 nM) under soil-based pot culture with EBL treatment solutions, and morphological, hormonal, and transcriptomic analyses were performed. Growth responses were non-monotonic, with BR400 producing the highest mean longitudinal leaf radius, petiole length, and leaf area, while transverse leaf radius was comparable to that under BR200; all four traits under BR400 were significantly greater than in the control. Hormone immunoreactive estimates varied across treatments, with the highest mean gibberellin (GA)- and brassinosteroid (BR)-immunoreactive estimates observed under BR400; however, neither differed significantly from CK. The highest mean indole-3-acetic acid (IAA)-immunoreactive estimate occurred under BR600. Transcriptomic analysis identified 1053 DEGs in leaves and 2028 in petioles under BR400 treatment compared with the control. GO and KEGG analyses revealed enrichment of cell-wall-organization- and phenylpropanoid biosynthesis-related categories among DEGs. Photosynthesis-related genes were down-regulated in leaves, accompanied by lower chlorophyll a and total chlorophyll concentrations under BR400. These findings characterize EBL-associated vegetative growth and transcriptional responses in *N. thermarum* and identify candidate genes and processes for future functional studies of water lily architecture.

## 1. Introduction

Water lilies (*Nymphaea* spp.), belonging to the family Nymphaeaceae, are perennial aquatic ornamentals renowned for their diverse floral colors, sacred cultural symbolism, and exceptional capacity for adsorbing eutrophic substances and harmful compounds from aquatic environments [1]. Leaf size varies markedly among water lilies, from the small leaves of miniature species to the exceptionally large floating leaves of the genus Victoria [2]. Large plant size can limit container cultivation and indoor ornamental use, making compact architecture a desirable trait. In this context, *Nymphaea thermarum*, discovered by German botanist Eberhard Fischer in 1987 [3], represents a unique genetic resource. Its exceptionally small stature makes *N. thermarum* a useful system for investigating hormonal regulation of vegetative growth and a potential genetic resource for breeding compact water lilies.

Plant stature is an important breeding trait, and phytohormones play central roles in its regulation [4]. Among phytohormones, brassinosteroids (BRs) are steroid hormones that regulate diverse developmental processes ranging from vegetative growth to reproductive development, including stem elongation, cell division, and vascular differentiation [5]. In model plants, BR perception by the BRI1/BAK1 receptor complex regulates BIN2 activity and the downstream transcription factors BZR1/BES1, which control genes involved in plant growth [6,7]. At the cellular level, BR promotes cell elongation by activating genes involved in cell wall biosynthesis and remodeling, including cellulose synthases (*CESA*), expansins (*EXP*), and xyloglucan endotransglucosylases/hydrolases (*XTH*), thereby remodeling cell wall composition and structure across species [8]. The mechanistic basis of BR-mediated cell expansion extends beyond transcriptional activation to cytoskeletal reorganization. In Arabidopsis pavement cells, BR signaling also influences cortical microtubule organization, providing another link between hormone signaling and cell growth [9].

Studies in model plants have shown that BR signaling interacts with auxin and gibberellin pathways through transcriptional regulators including BZR1, PIF4, and ARF6 [10]. For instance, the effects of exogenous BR can depend on concentration, as reported for drought-related responses in melon [11]. In wheat, reduced BR signaling can produce semi-dwarf architecture and improve grain yield, illustrating the relevance of BR signaling to crop architecture [12]. Similarly, the deployment of BR-related dwarfing alleles in barley exemplifies their application in cereal improvement, underscoring the role of BR signaling in crop improvement [13]. Despite these advances in model plants, BR-mediated regulation of plant stature in aquatic ornamental species remains largely unexplored, representing a significant gap in our understanding of plant architecture regulation. However, dose-dependent growth responses to EBL and the associated hormonal and tissue-specific transcriptional changes remain insufficiently characterized in *N. thermarum*. Comparing leaf and petiole responses may help identify shared and organ-specific processes associated with EBL-induced growth.

This study examined dose-dependent vegetative growth responses to exogenous EBL and the associated hormonal and transcriptional changes in *N. thermarum*. Specifically, we assessed growth traits, photosynthetic pigment concentrations, and leaf hormone immunoreactive estimates across an EBL concentration gradient. RNA-seq comparisons were restricted to BR400 and CK to characterize the transcriptional response associated with the selected growth-promoting concentration rather than the complete dose–response curve. We hypothesized that (i) EBL alters vegetative organ growth in a concentration-dependent manner, (ii) these growth responses are accompanied by changes in leaf hormone immunoreactive estimates, and (iii) leaf and petiole responses involve shared and tissue-specific transcriptional changes in growth-related processes, including cell wall remodeling. The results provide a basis for investigating BR-associated growth regulation and evaluating candidate genes relevant to water lily architecture.

## 2. Results

### 2.1. Dose-Dependent Effects of Exogenous EBL on Plant Architecture of N. thermarum

**Figure 1:**
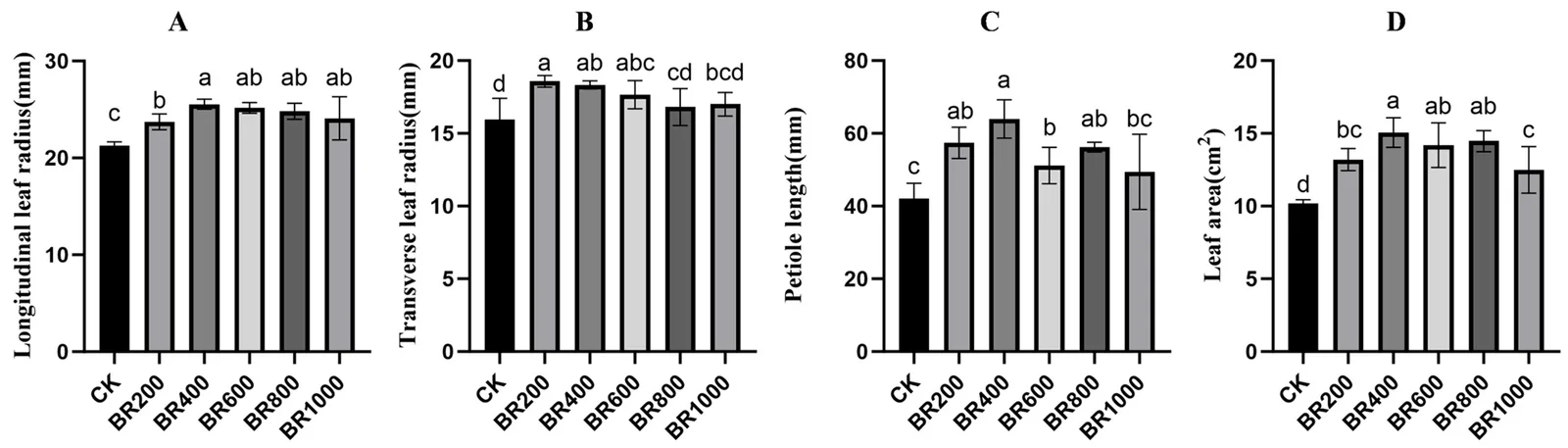
Dose–response effects of 24-epibrassinolide (EBL) on the plant architecture of *Nymphaea thermarum*. Plants were treated with EBL at concentrations of 0 (CK), 200, 400, 600, 800, and 1000 nM under soil-based pot culture with EBL treatment solutions for 3 weeks. (**A**) Longitudinal leaf radius; (**B**) transverse leaf radius; (**C**) petiole length; (**D**) leaf area. Data are means of four biological replicates; different lowercase letters above the bars indicate significant differences among treatments (one-way ANOVA followed by Waller–Duncan multiple range test with a K-ratio of 100). Error bars represent ± SD. Sensitivity analyses did not support the leaf-length differences between BR1000 and CK or between BR400 and BR200, or the leaf-area differences between BR600 and CK, BR1000 and CK, BR400 and BR200, BR400 and BR1000, BR600 and BR1000, or BR800 and BR1000 (Supplementary Table S16).

**Figure 2:**
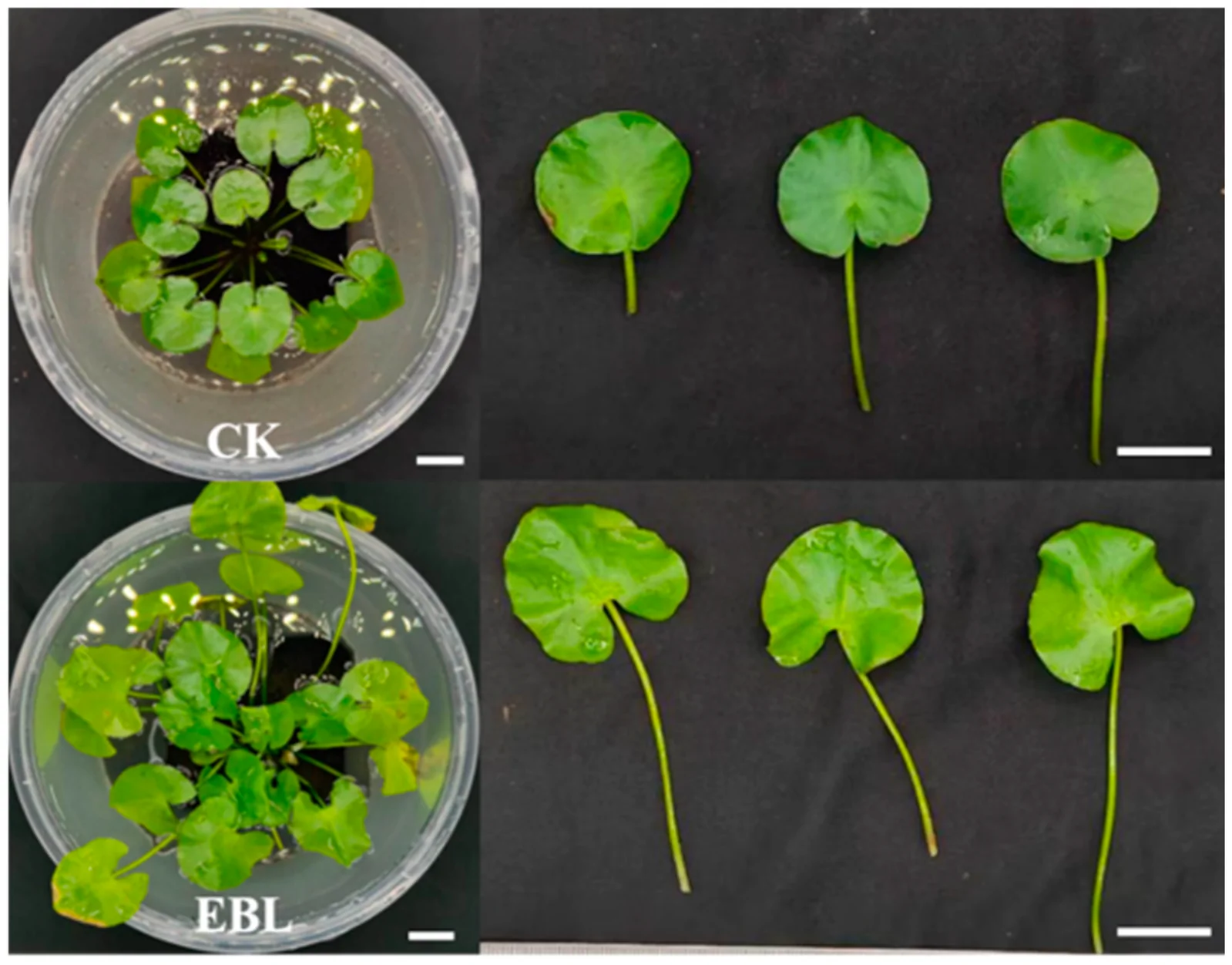
Comparison of whole-plant phenotype and leaf morphology of *Nymphaea thermarum* under 400 nM EBL (BR400) treatment versus the control (CK). Scale bar = 2 cm.

To elucidate the regulatory effects of exogenous brassinosteroid (BR) on vegetative growth of *N. thermarum*, the world’s smallest water lily, plants were subjected to a concentration gradient of 24-epibrassinolide (EBL) at 0, 200, 400, 600, 800, and 1000 nM under soil-based pot culture with EBL treatment solutions. Morphological parameters were systematically measured after 3 weeks of treatment. The results demonstrated that EBL application significantly affected longitudinal leaf radius, transverse leaf radius, petiole length, and leaf area. Growth responses varied among traits and were non-monotonic across the tested concentrations (Figure 1). Longitudinal leaf radius and leaf area were significantly greater under BR400 than BR200, but these differences were not supported by the sensitivity analyses (Supplementary Table S16). Petiole length was significantly greater under BR400 than BR600. The remaining comparisons between BR400 and these neighboring treatments were not significant. BR400 was selected for RNA-seq because it produced the highest mean longitudinal leaf radius, petiole length, and leaf area, together with an increase in transverse leaf radius relative to CK. Compared with CK, BR400-treated plants displayed larger leaf blades and longer petioles, consistent with the quantitative measurements (Figure 2).

### 2.2. Effects of Exogenous EBL on Chlorophyll Content

**Figure 3:**
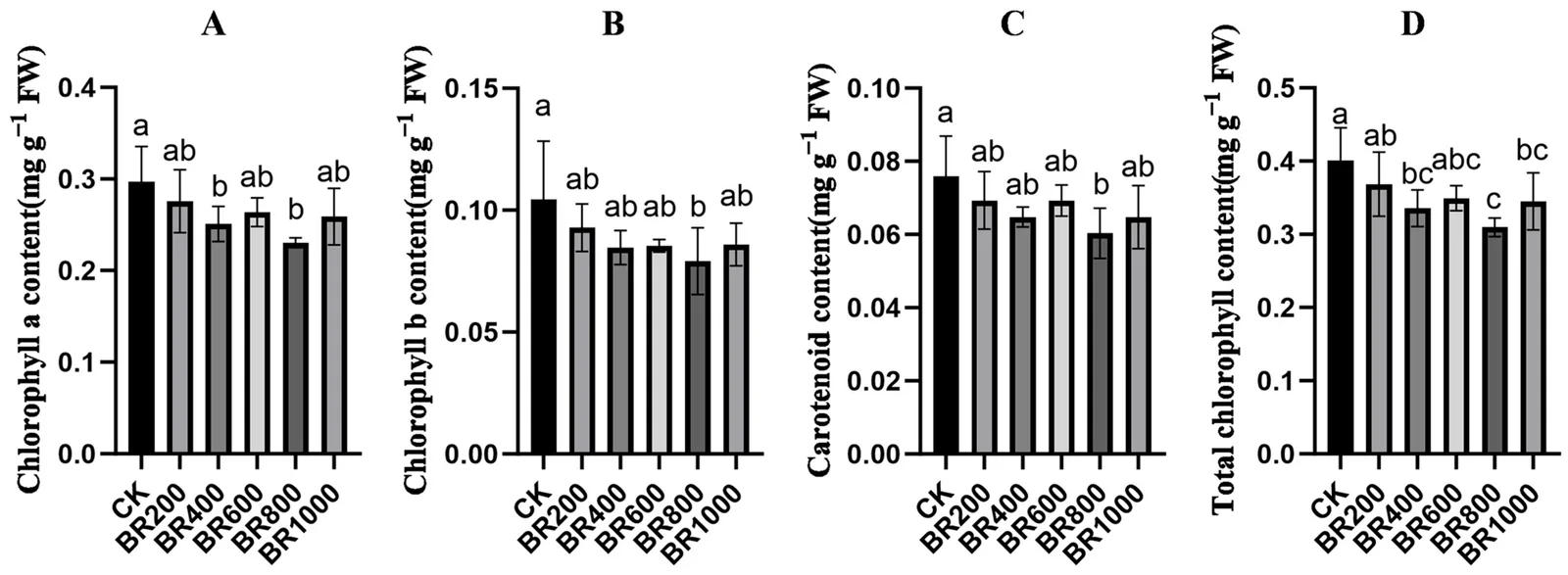
Effects of exogenous 24-epibrassinolide (EBL) on photosynthetic pigment contents in *N. thermarum* leaves. (**A**) Chlorophyll a content; (**B**) chlorophyll b content; (**C**) carotenoid content; (**D**) total chlorophyll content. FW, fresh weight. Data are means of four biological replicates; different lowercase letters above the bars indicate significant differences among treatments (one-way ANOVA followed by Waller–Duncan multiple range test with a K-ratio of 100). Error bars represent ± SD. The differences between BR800 and CK in chlorophyll b and carotenoid concentrations were not supported by the sensitivity analyses (Supplementary Table S16).

To investigate whether BR-promoted vegetative growth was associated with alterations in photosynthetic pigment metabolism, chlorophyll a, chlorophyll b, carotenoid, and total chlorophyll contents were quantified at 21 d post-treatment (Figure 3). Compared with CK, chlorophyll a concentrations were significantly lower under BR400 and BR800, and total chlorophyll concentrations were significantly lower under BR400, BR800, and BR1000. Chlorophyll b and carotenoid concentrations were significantly lower only under BR800; however, these differences were not supported by the sensitivity analyses (Supplementary Table S16).

### 2.3. Effects of Exogenous EBL on Leaf Hormone Measurements

**Figure 4:**
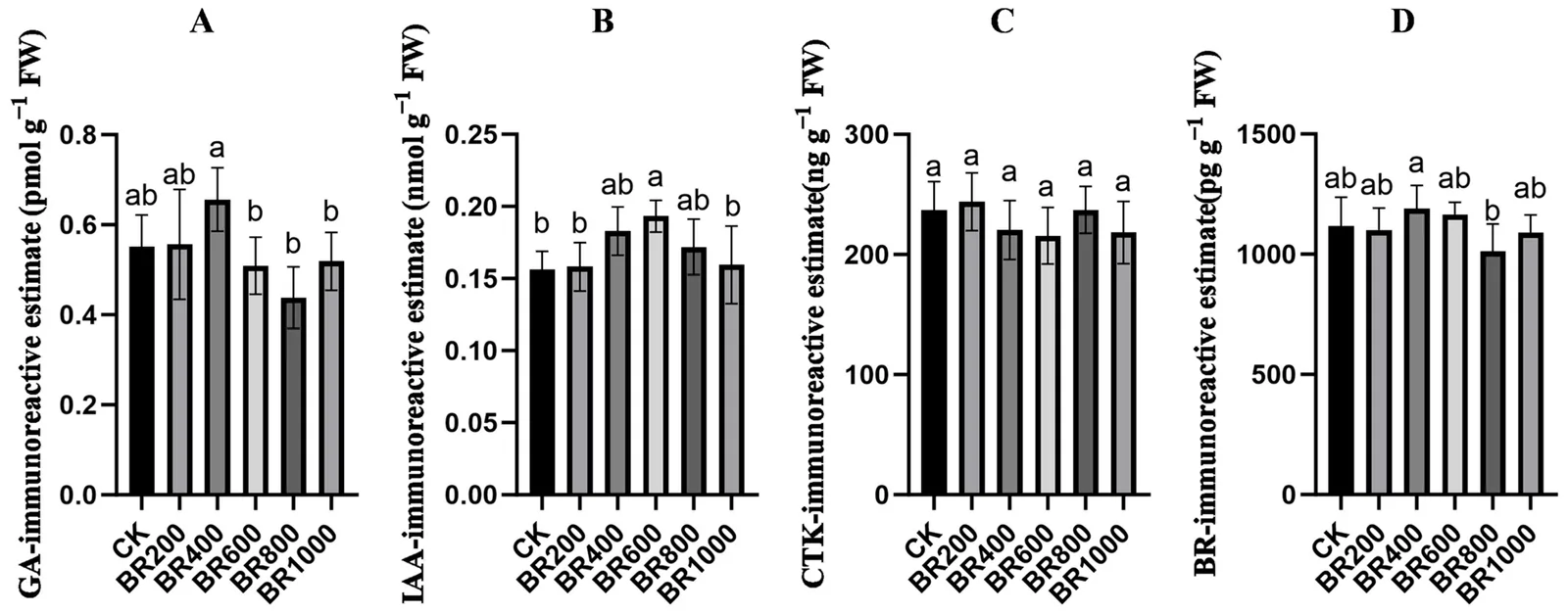
Effects of exogenous 24-epibrassinolide (EBL) on hormone immunoreactive estimates in *N. thermarum* leaves. (**A**) GA-immunoreactive estimate; (**B**) IAA-immunoreactive estimate; (**C**) CTK-immunoreactive estimate; (**D**) BR-immunoreactive estimate. Data are means of four biological replicates; different lowercase letters above the bars indicate significant differences among treatments (one-way ANOVA followed by Waller–Duncan multiple range test with a K-ratio of 100). Error bars represent ± SD.

To investigate the dose-dependent treatment-associated variation in leaf hormone immunoreactive estimates in *N. thermarum* under exogenous EBL treatment and their association with phenotypic alterations, leaf GA-, IAA-, CTK-, and BR-immunoreactive estimates were measured after 3 weeks of EBL treatment (Figure 4). The highest mean GA- and BR-immunoreactive estimates occurred under BR400, whereas the highest mean IAA-immunoreactive estimate occurred under BR600. BR400 had a higher GA-immunoreactive estimate than BR600, BR800, and BR1000, but belonged to a common homogeneous subset with CK and BR200. The IAA-immunoreactive estimate under BR600 was higher than those under CK, BR200, and BR1000. BR400 had a higher BR-immunoreactive estimate than BR800, but belonged to a common subset with CK. All CTK-immunoreactive estimates belonged to the same homogeneous subset. These measurements represent assay-derived immunoreactive estimates rather than chemically verified endogenous hormone concentrations.

### 2.4. Transcriptome Data Quality Assessment

To characterize transcriptional responses associated with EBL treatment, RNA-seq was performed on leaf and petiole tissues from BR400-treated and control plants. The design comprised four sample groups, each with four biological replicates, yielding 16 libraries. Mean clean-data yields were 6.73 Gb for leaves and 6.46 Gb for petioles. Q30 percentages ranged from 97.79% to 98.19% in leaves and from 96.74% to 96.91% in petioles. The arithmetic mean total mapping rates across libraries were 96.88% and 96.50%, respectively. Per-library sequencing and alignment statistics, including sample names and library identifiers, are provided in Supplementary Table S17.

**Figure 5:**
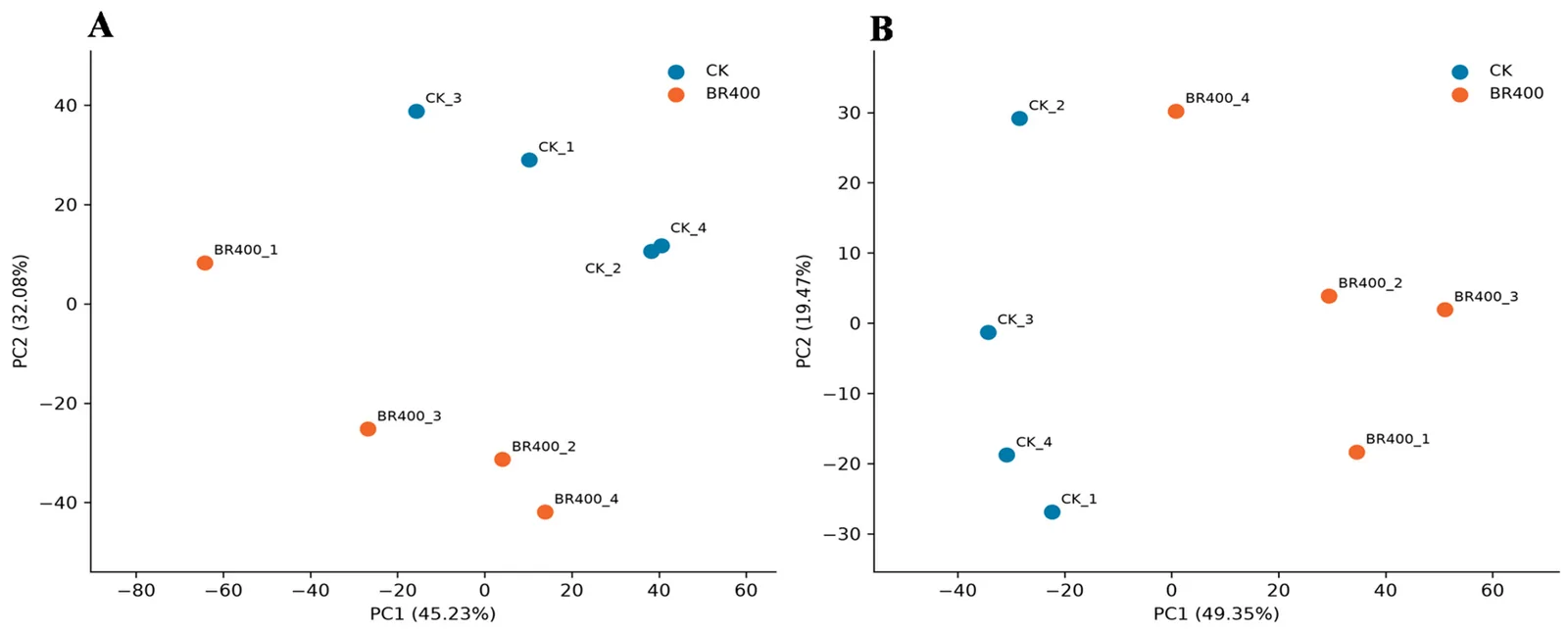
Principal component analysis (PCA) of RNA-seq data from *N. thermarum* leaf and petiole tissues under BR400 treatment and control (CK). (**A**) Leaves; (**B**) petioles. Each point represents one biological replicate (n = 4 per treatment within each tissue). PCA was performed separately for each tissue using the 500 most variable genes after variance-stabilizing transformation of raw counts. Genes with fewer than 10 total counts across the eight samples within a tissue were excluded before transformation. Percentages on the axes indicate the variance explained by each principal component. All biological replicates were retained, and confidence ellipses were not plotted. CK, control; BR400, 400 nM 24-epibrassinolide treatment.

Principal component analysis (PCA) was performed separately for leaves and petioles using variance-stabilized RNA-seq counts and the 500 most variable genes within each tissue (Figure 5). In leaves, PC1 and PC2 explained 45.23% and 32.08% of the variance, respectively; the corresponding values in petioles were 49.35% and 19.47%. The sample distributions showed treatment-associated differences alongside within-group dispersion in both tissues. Inspection of the quality summaries identified a lower GC percentage and total mapping rate for leaf sample BR400_4 (42.63% and 93.05%, respectively), although its Q30 percentage was 98.19%. These differences do not establish the cause of the within-group PCA dispersion. All biological replicates were retained.

### 2.5. Differential Expression Gene (DEG) Analysis

**Figure 6:**
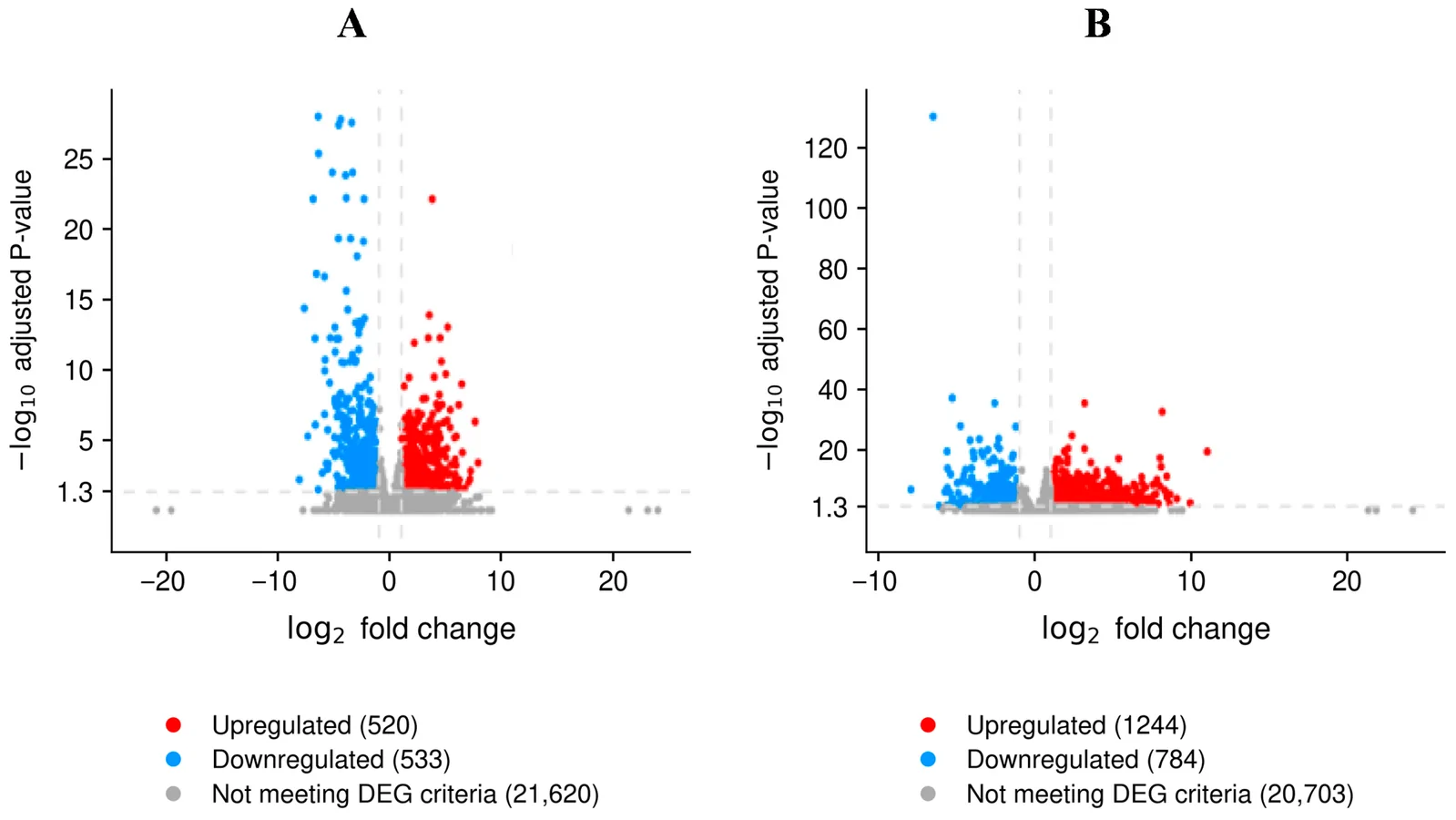
Volcano plot visualization of differentially expressed genes (DEGs) in *Nymphaea thermarum* under BR400 treatment compared with control (CK). (**A**) Leaf DEGs; (**B**) petiole DEGs. The x-axis represents log_2_(fold change) and the y-axis represents −log_10_(adjusted *p*-value). Red dots indicate significantly up-regulated genes, blue dots indicate significantly down-regulated genes, and gray dots indicate non-significantly differentially expressed genes.

Differential expression analysis identified 1053 DEGs in leaves, including 520 upregulated and 533 downregulated genes, and 2028 DEGs in petioles, including 1244 upregulated and 784 downregulated genes (Figure 6). DEGs were defined using adjusted *p* ≤ 0.05 and |log_2_ fold change| ≥ 1. Complete DEG lists for leaves and petioles, including gene identifiers, per-sample FPKM values, log2 fold changes, raw and adjusted *p*-values, and functional annotations, are provided in Supplementary Tables S1 and S2, respectively.

Cross-tissue comparison identified 512 shared DEGs. Among these, 255 were upregulated, and 248 were downregulated in both tissues. Five genes were upregulated in leaves but downregulated in petioles, whereas four showed the opposite pattern. An additional 541 DEGs were detected only in leaves and 1516 only in petioles under the applied thresholds. Expression values, differential expression statistics, and annotations for these shared DEGs are provided in Supplementary Table S3. RT-qPCR analysis of five selected genes showed increased expression of EXPA8-like and PAL1-like and decreased expression of XTH7-like and CPD/CYP90A1-like in both leaves and petioles under BR400, consistent with the directions observed in RNA-seq. DWF4/CYP90B1-like was also downregulated in both tissues by RT-qPCR, although it met the RNA-seq DEG criteria only in petioles (Supplementary Figure S1).

### 2.6. GO Enrichment Analysis

**Figure 7:**
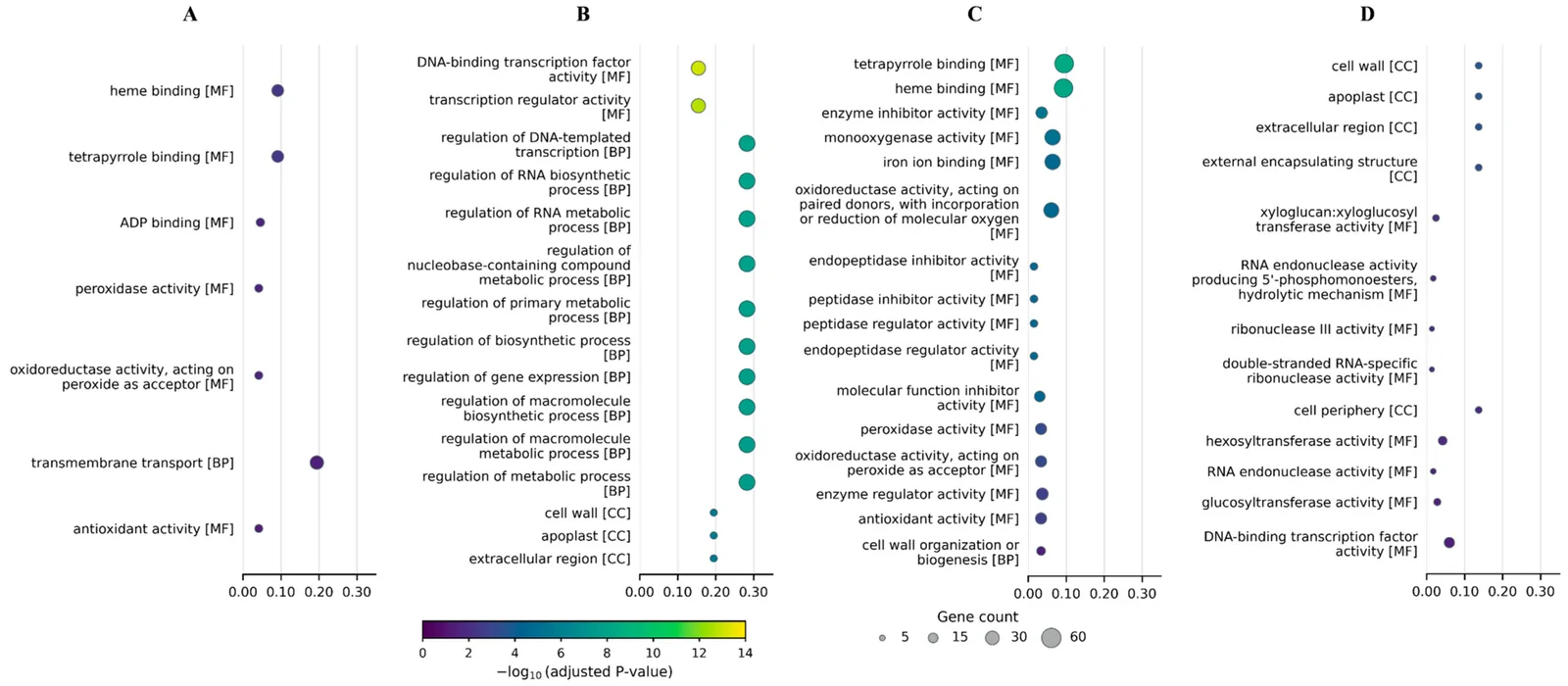
GO enrichment of upregulated and downregulated genes in *N. thermarum* leaves and petioles following BR400 treatment. (**A**) Upregulated leaf DEGs; (**B**) downregulated leaf DEGs; (**C**) upregulated petiole DEGs; (**D**) downregulated petiole DEGs, relative to CK. DEGs were defined by adjusted *p* ≤ 0.05 and |log_2_ fold change| ≥ 1. Only GO terms with adjusted *p* < 0.05 are shown. Terms are ordered by increasing adjusted *p*. Up to 15 significant GO terms are shown per panel, except panel C, which additionally includes cell wall organization or biogenesis (GO:0071554), resulting in 16 terms. GeneRatio denotes the proportion of annotated input DEGs associated with each GO term within the corresponding GO category. Dot size indicates the number of DEGs associated with each term, and color indicates −log_10_(adjusted *p*). BP, biological process; CC, cellular component; MF, molecular function. Complete enrichment results are provided in the supplementary tables.

GO enrichment analysis was performed separately for upregulated and downregulated DEGs in each tissue, with adjusted *p* < 0.05 used as the significance threshold. In leaves, upregulated DEGs were enriched in terms including transmembrane transport, heme binding, peroxidase activity, and antioxidant activity. Downregulated leaf DEGs were enriched in transcriptional regulation-related terms, cell wall, and apoplast (Figure 7A,B).

In petioles, upregulated DEGs were significantly enriched in response to oxidative stress, sulfur compound metabolic process, transmembrane transport, and cell wall organization or biogenesis (Supplementary Table S11). Downregulated petiole DEGs were enriched in cell wall, apoplast, and xyloglucan transferase activity (Figure 7C,D). These results indicate tissue- and direction-dependent enrichment patterns among EBL-responsive DEGs. Figure 7 displays up to 15 significant terms per panel, except panel C, which includes one additional cell wall-related term. Complete GO enrichment outputs for all, upregulated, and downregulated DEGs are provided in Supplementary Tables S4–S6 for leaves and Tables S10–S12 for petioles, respectively.

### 2.7. KEGG Pathway Enrichment Analysis

**Figure 8:**
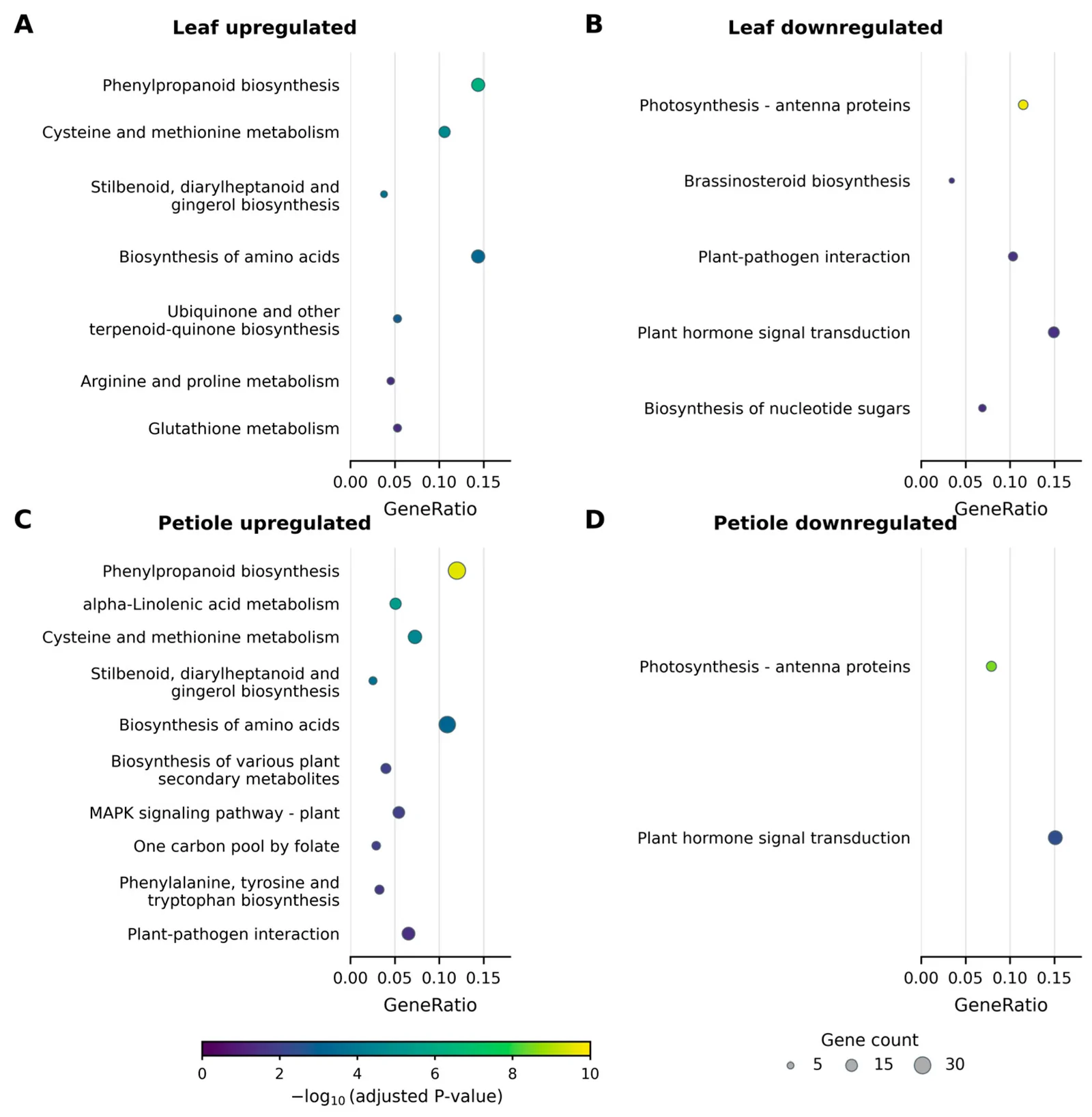
KEGG pathway enrichment of upregulated and downregulated genes in *N. thermarum* leaves and petioles following BR400 treatment. (**A**) Upregulated leaf DEGs; (**B**) downregulated leaf DEGs; (**C**) upregulated petiole DEGs; (**D**) downregulated petiole DEGs, relative to CK. DEGs were defined by adjusted *p* ≤ 0.05 and |log_2_ fold change| ≥ 1. All pathways with adjusted *p* < 0.05 are shown, ordered by increasing adjusted *p*. GeneRatio denotes the proportion of annotated input DEGs associated with each KEGG pathway. Dot size indicates the number of DEGs associated with each pathway, and color indicates −log_10_(adjusted *p*). Complete enrichment results are provided in the supplementary tables.

KEGG enrichment analysis identified phenylpropanoid biosynthesis (ath00940) among the significantly enriched pathways in upregulated DEGs from both leaves and petioles, involving 19 and 33 genes, respectively. Photosynthesis–antenna proteins (ath00196) was significantly enriched in downregulated DEGs from both tissues, involving 10 genes in leaves and 11 in petioles (Figure 8).

Plant hormone signal transduction (ath04075) was also significantly enriched in the downregulated DEG sets of both tissues. Brassinosteroid biosynthesis (ath00905) was also significantly enriched among downregulated leaf DEGs (adjusted *p* = 0.0339; Figure 8B; Supplementary Table S9). The contributing genes were two putative CPD/CYP90A1 homologs (EJ110_NYTH45884 and EJ110_NYTH21999) and one putative BAS1/CYP734A1 homolog (EJ110_NYTH46156). In petioles, additional pathways enriched among upregulated DEGs included alpha-linolenic acid metabolism and MAPK signaling pathway–plant. Complete KEGG enrichment outputs for all, upregulated, and downregulated DEGs are provided in Supplementary Tables S7–S9 for leaves and Tables S13–S15 for petioles, respectively.

## 3. Discussion

Exogenous EBL increased leaf dimensions and petiole length in *N. thermarum*. BR400 produced the highest mean longitudinal leaf radius, petiole length, and leaf area, whereas the highest mean transverse leaf radius occurred under BR200. These results demonstrate that vegetative organ size in this miniature water lily remains responsive to exogenous BR treatment. Genetic studies in *Arabidopsis* established the involvement of the receptor kinase BRI1 in BR signaling and showed that nuclear accumulation of BES1 connects BR perception with gene expression and stem elongation [14,15]. Subsequent identification of BZR1 target genes revealed a transcriptional network linking BR signaling to multiple growth-related processes [7]. This framework provides a biological context for the observed growth response, but increased organ size following EBL treatment does not establish that the intrinsic dwarf stature of *N. thermarum* results from deficient BR biosynthesis or signaling. Resolving the basis of its small stature would require comparisons of endogenous BR profiles, signaling activity, and cellular growth with those of larger water lilies.

Mean longitudinal leaf radius, petiole length, and leaf area were lower at concentrations above BR400, although not all comparisons with BR400 were significant. BR signaling includes feedback control: BZR1 can repress BR biosynthetic genes while regulating downstream growth responses [16]. In the present study, two putative CPD/CYP90A1 homologs (EJ110_NYTH45884 and EJ110_NYTH21999) were downregulated in leaves. EJ110_NYTH45884 was also downregulated in petioles, together with a putative DWF4/CYP90B1 homolog (EJ110_NYTH13790). These changes are consistent with feedback repression of BR biosynthesis following EBL treatment, although BZR1 activity was not directly assessed. BAS1/CYP734A1-like genes, which participate in BR inactivation, showed divergent responses: EJ110_NYTH46156 was downregulated, whereas EJ110_NYTH53828 was upregulated in both tissues. Comparisons with carrot are relevant because exogenous BR has been associated with petiole elongation, developmental changes in BR-related gene expression, and changes in cell length, GA content, and cellulose deposition [17,18]. These studies support the potential for BR to alter petiole growth across species while also illustrating the value of combining organ measurements with anatomical and biochemical evidence. BR400 produced the highest mean longitudinal leaf radius, petiole length, and leaf area within the tested concentration range, but should not be regarded as a general optimum for *N. thermarum* cultivation. The weaker responses at higher concentrations should also be distinguished from inhibition relative to CK.

The transcriptome results suggest that EBL-associated growth involved changes in cell wall-related gene expression rather than uniform activation of a single wall biosynthetic pathway. The enrichment of cell wall organization or biogenesis among upregulated petiole DEGs supports the involvement of wall-related transcriptional processes in the EBL response (Supplementary Table S11) but does not demonstrate increased wall biosynthesis or altered wall composition. Downregulated DEGs in both tissues were enriched in cell wall and apoplast (Figure 7B,D). In Arabidopsis roots, single-cell analysis showed that BR-responsive transcription varies with cell identity and developmental stage and linked the transition toward elongation in the cortex to increased expression of cell wall-related genes [19]. Spatial regulation of BR biosynthesis also contributes to optimal root growth [20]. These findings provide a comparative framework for investigating EBL-associated growth in *N. thermarum*. However, bulk RNA-seq cannot distinguish changes within individual cell types from differences in developmental state or tissue composition. The wall-related DEGs identified here therefore provide candidates for functional and anatomical studies of organ expansion.

BR regulation of cell walls has been demonstrated directly in other systems. In *Arabidopsis*, altered BR signaling affects CESA expression and cellulose biosynthesis [21], while the carrot study linked BR-promoted petiole elongation with changes in cellulose deposition and cell length [18]. The present data extend the comparison to a miniature aquatic plant but do not demonstrate the same biochemical outcome. Phenylpropanoid biosynthesis was enriched among upregulated DEGs in both leaves and petioles (Figure 8), suggesting that EBL treatment was associated with changes in this metabolic pathway. An EBL-associated phenylpropanoid response has also been reported in watermelon under zinc stress [22]. However, that study addressed stress tolerance, whereas the present experiment examined vegetative growth; the physiological roles of the transcriptional changes may therefore differ. Because the phenylpropanoid pathway contains branches contributing to several classes of metabolites, pathway enrichment alone cannot establish increased lignin accumulation. Measurements of cellulose, lignin, relevant metabolites, and wall mechanical properties would help determine whether these changes contribute to organ expansion, structural support, or other functions.

Leaves and petioles exhibited a shared component of the EBL response alongside differences in DEG detection and enrichment. Of the 512 shared DEGs, 503 changed in the same direction, and nine showed opposite responses. The predominance of concordant changes indicates that part of the response was shared between the two organs, while the discordant genes warrant further investigation as candidates for tissue-dependent regulation. The larger number of DEGs in petioles should not be interpreted by itself as evidence of greater BR sensitivity: DEG detection also depends on baseline expression, biological variation, and the statistical threshold. Moreover, genes meeting the DEG threshold in only one tissue do not necessarily show a significant difference in treatment response between tissues. In *Arabidopsis*, auxin and BR contribute to petiole elongation under shade [23], providing a precedent for hormonal regulation of this organ. The present experiment did not manipulate light conditions, so that mechanism remains a comparative framework rather than an explanation established for *N. thermarum*. Testing treatment-by-tissue interactions and examining the shared genes at cellular resolution would clarify the common and organ-dependent components of the response.

Leaf hormone immunoreactive estimates varied among treatments, although their patterns did not fully coincide with the morphological responses. The highest mean GA and BR estimates occurred under BR400, whereas the highest mean IAA estimate occurred under BR600. Neither the GA nor the BR estimate under BR400 differed from CK, whereas the IAA estimate under BR600 was higher than CK. Molecular studies have identified several points of interaction among these pathways: ARF2 integrates auxin and BR signaling [24], DELLA proteins interact with BZR1 to connect GA and BR responses [25,26], and ARF6, BZR1, and PIF4 cooperate in a growth-regulatory circuit whose DNA-binding activities are inhibited by RGA [27]. These findings offer plausible mechanisms through which hormone pathways can coordinate cell elongation, but bulk hormone immunoreactive estimates cannot identify which mechanism operated in the present experiment. In addition, enrichment of plant hormone signal transduction among downregulated DEGs in both tissues indicates that hormone-related transcriptional changes cannot be summarized as uniform pathway activation. All four hormone measurements should be interpreted as immunoreactive estimates because spike-and-recovery, dilution-linearity, and matrix-effect validation were not performed for *N. thermarum* leaf extracts. The BR estimate requires additional caution because the manufacturer reported 4.37% cross-reactivity with epibrassinolide. Although leaves were rinsed before extraction, contributions from absorbed EBL cannot be excluded. The relatively high CTK values should be interpreted as zeatin-equivalent immunoreactive estimates rather than chemically verified endogenous zeatin concentrations, given the uncharacterized cross-reactivity with other cytokinins and the absence of leaf-matrix validation and low-range precision data. No treatment differences were detected in the CTK-immunoreactive estimate at day 21; this finding is limited to the sampled tissue and time point and does not exclude cytokinin involvement in growth regulation.

Treatment-associated reductions in selected pigment concentrations, together with enrichment of photosynthesis–antenna proteins among downregulated DEGs, suggest changes in aspects of the photosynthetic apparatus alongside organ growth. Under BR400, chlorophyll a and total chlorophyll concentrations were lower than CK, whereas no differences from CK were detected for chlorophyll b or carotenoids. BR and environmental signals converge at several regulatory points in *Arabidopsis*: BZR1 interacts with PIF4 to integrate hormonal and environmental responses [28], GATA2 participates in BR–light signaling interactions [29], and phytochrome A represses the BR pathway during prolonged strong shade [30]. These studies demonstrate links between BR signaling and light-responsive growth, but they do not establish increased shade tolerance or reduced photosynthetic performance in the present experiment. Indeed, EBL enhanced photosynthetic capacity in cucumber in a study that directly measured gas exchange, chlorophyll fluorescence, and Rubisco activity [31]. This comparison emphasizes that pigment concentrations and transcript abundance should not be treated as substitutes for physiological measurements. Here, lower pigment concentrations per unit fresh mass may partly reflect tissue expansion or changes in tissue composition. A growth–photosynthesis trade-off therefore remains untested. Gas exchange, fluorescence, and pigment measurements per unit leaf area, combined with a time course, would clarify whether the transcriptional changes were accompanied by altered light harvesting or carbon assimilation. RT-qPCR supported the expression changes in selected genes, but validation was restricted to BR400 versus CK and did not cover the full EBL concentration gradient.

**Figure 9:**
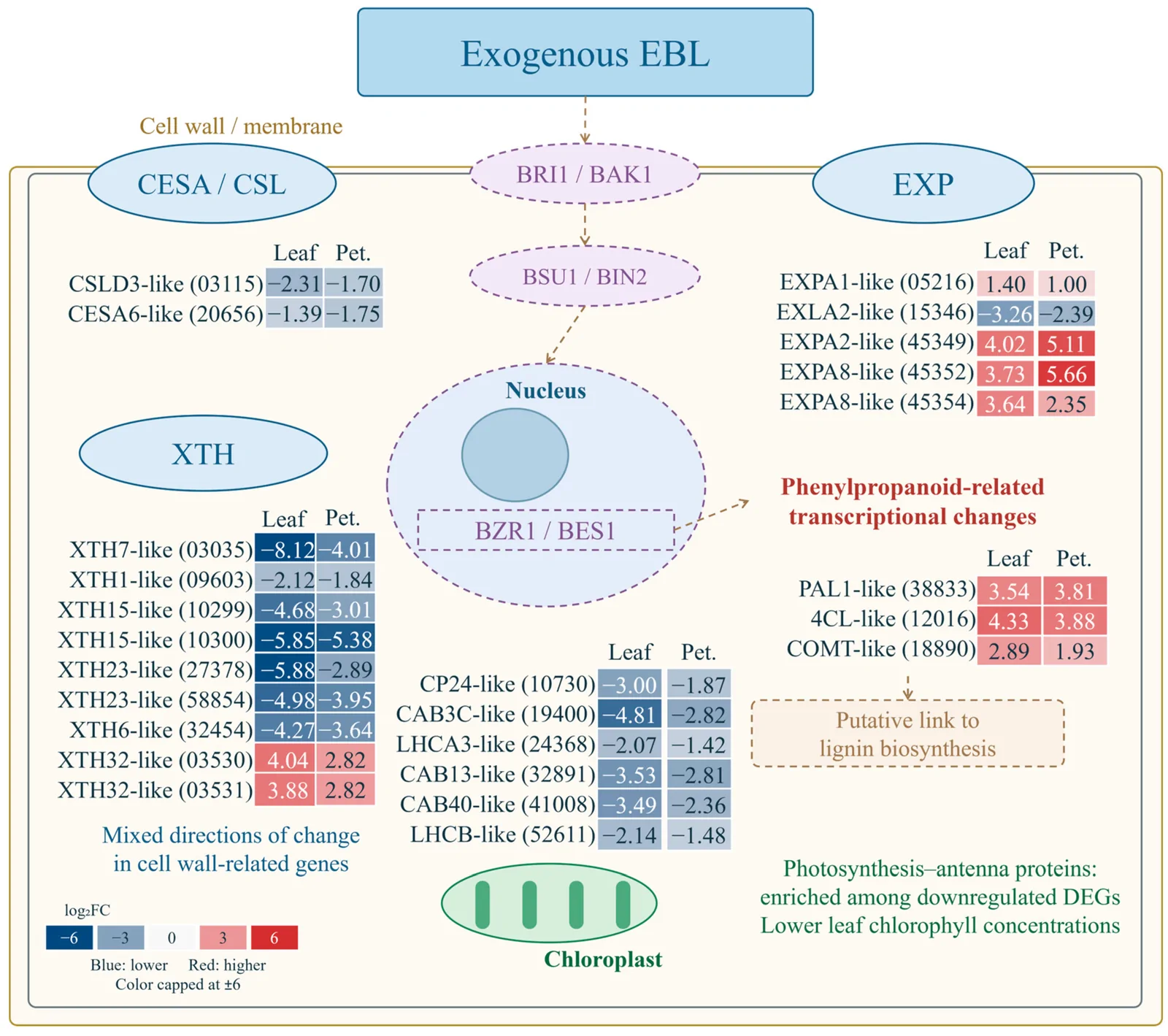
Hypothetical working model of the EBL-associated growth response in *Nymphaea thermarum.* Heatmaps show log_2_ fold changes for selected shared DEGs in leaves (Leaf) and petioles (Pet.) under BR400 relative to CK. Red and blue indicate upregulation and downregulation, respectively; color saturation is capped at ±6. Parenthetical numbers denote gene identifier suffixes following gene_EJ110_NYTH, and gene names indicate putative homologs. Solid blue outlines identify transcriptome-supported gene groups; dashed purple outlines and brown arrows denote unvalidated signaling components and proposed regulatory links.

The application of these findings depends on the desired plant architecture. Research in wheat demonstrated that attenuating BR signaling can improve grain yield in a particular semi-dwarf genetic background [12], illustrating that favorable outcomes do not necessarily require stronger BR signaling or larger organs. In *N. thermarum*, the present EBL treatment increased vegetative organ size and is therefore most relevant to objectives favoring increased growth rather than further miniaturization. Whether this response improves ornamental value will depend on its persistence, effects on flowering, and compatibility with cultivation conditions. The hypothetical working model summarizes selected transcriptional responses associated with EBL treatment and distinguishes these observations from literature-based regulatory hypotheses (Figure 9). The contrasting expression patterns within cell wall-related gene groups suggest a heterogeneous transcriptional response rather than uniform activation of wall biosynthesis. Possible links between BR signaling, these transcriptional changes, and organ growth require functional validation; neither enhanced lignin accumulation nor carbon reallocation was demonstrated. Several limitations should be considered when interpreting these findings. RNA-seq and physiological measurements were obtained at a single endpoint, 21 days after treatment. The transcriptional profiles therefore cannot distinguish early, direct responses to EBL from subsequent developmental changes or homeostatic adjustments, and time-course studies are needed to resolve their temporal relationships. Hormone measurements were restricted to leaves, and the ELISA assays were not validated for recovery, dilution linearity, or matrix effects in *N. thermarum* extracts. Although the manufacturer reported 4.37% cross-reactivity of the BR assay with epibrassinolide, its contribution to the measured BR-immunoreactive estimates was not determined. Cross-species annotations provide putative functional assignments, while the absence of direct cell wall, anatomical, and photosynthetic measurements limits mechanistic interpretation. Relative humidity, photoperiod, treatment-solution pH and electrical conductivity, and photosynthetic photon flux density were not monitored, limiting environmental characterization of the experiment. The pots were neither rotated nor arranged in blocks during the experiment, so positional effects cannot be excluded despite random assignment of plants to treatments. Within these constraints, the study identifies an EBL-responsive growth phenotype and candidate transcriptional processes for further functional and physiological investigation.

## 4. Materials and Methods

### 4.1. Plant Materials and Culture Conditions

One-year-old *Nymphaea thermarum* seedlings were obtained from the Water Lily Sub-garden of the Hainan Tropical Herbaceous Flower Germplasm Repository, Sanya, Hainan, China. Healthy plants with uniform growth and no visible pests or diseases were selected. Each plant was cultivated in a 12 cm-diameter plastic inner pot containing water lily mud, an aquatic plant substrate, and placed inside a 20 cm-diameter plastic outer container. The outer container was a truncated cone, 9.5 cm in height, with bottom and top diameters of 12.5 and 20 cm, respectively. A seedling pot containing water lily mud was placed inside each outer container. Each container received 1 L of treatment solution, with no pre-existing standing water. The nominal EBL concentrations (200–1000 nM) were calculated based on this final solution volume. Plants were maintained under natural light and naturally ventilated conditions at temperatures of 25–35 °C. The recorded illuminance was approximately 12,000 lx. Treatment solutions were not actively aerated. One-year-old plants with uniform growth, healthy appearance, and no visible pests or diseases were selected. Four biological replicates were used per treatment, with one plant per replicate.

### 4.2. Exogenous BR Treatment Experimental Design

The experiment was conducted from 2 to 23 January 2026 at the Sanya Institute of Nanjing Agricultural University. 24-Epibrassinolide (EBL) was used to prepare the hormone solutions. 24-Epibrassinolide (EBL) was purchased from Shanghai Yuanye Bio-Technology Co., Ltd. (Shanghai, China; catalog no. S18014; purity: 92%). EBL was first dissolved in a small volume of DMSO to prepare a stock solution, which was then diluted with deionized water to the working concentrations. The final DMSO concentration was 0.1% (*v*/*v*) in all treatment and control solutions. The control (CK) solution consisted of deionized water containing the same DMSO concentration (0.1%, *v*/*v*) as the EBL treatment solutions. Six concentration treatments were established: 0 (control, CK), 200, 400, 600, 800, and 1000 nM. Twenty-four plants were randomly assigned to the six treatments, with four biological replicates per treatment and one plant representing one biological replicate. Pots remained in their assigned positions and were not rotated during the experiment. Each outer container received 1 L of the corresponding treatment solution, and the ambient growth temperature was maintained at 25–35 °C. The solutions were replaced every 3 days with freshly prepared EBL solutions, and the control was replaced with deionized water containing 0.1% (*v*/*v*) DMSO. Each treatment had four biological replicates, and the treatment lasted for 3 weeks. The largest fully expanded leaf from each plant was selected for sampling. Leaf samples were used for pigment, hormone, and transcriptome analyses, and petiole samples were collected from the corresponding leaf. All samples were collected between 9:00 and 10:00 a.m. Samples for pigment determination, hormone assays, and RNA-seq were collected at the same sampling time from the same plant within each biological replicate.

### 4.3. Morphological Parameter Measurement

Newly emerging leaves were tagged at the beginning of treatment. At day 21, the largest fully expanded leaf that had developed during the treatment period was selected from each plant. The selected leaf was used for morphological measurements and sampling for pigment, hormone, and transcriptome analyses, with the corresponding petiole collected where required. Longitudinal leaf radius (from the petiole attachment point to the leaf tip), transverse leaf radius (from the petiole attachment point to the leaf margin at the widest part of the blade), and petiole length (from the base to the leaf attachment) were measured using an electronic digital caliper. Single-leaf area was measured directly using a portable leaf area meter (CID, CI-202), rather than calculated from the linear measurements or summed across multiple leaves. Each plant was measured three times (technical replicates), and the mean value of each plant was used as one biological replicate in the subsequent statistical analysis.

### 4.4. Photosynthetic Pigment Determination

Leaf samples were collected at 21 d after treatment, and photosynthetic pigment contents were determined by the 95% ethanol extraction-spectrophotometric method. Harvested leaves were immediately rinsed three times with deionized water to minimize residual treatment solution on the leaf surface and blotted dry with filter paper before pigment extraction. Fresh leaf tissues (0.1 g, midribs removed) were cut into small pieces and placed in test tubes. Five milliliters of 95% ethanol were added to each tube to completely immerse the samples, followed by incubation in the dark for 24 h. The absorbance of the extract was measured at 665 nm, 649 nm, and 470 nm using a spectrophotometer (Tecan, Männedorf, Switzerland), with 95% ethanol as the blank. Absorbance at each wavelength was measured in triplicate for each extract, and the mean value was used to calculate pigment contents. The concentrations of chlorophyll a (Ca), chlorophyll b (Cb), and carotenoids (Cx.c) were calculated using the following equations:

Ca = 13.95 × A665 − 6.88 × A649

Cb = 24.96 × A649 − 7.32 × A665

Cx.c = (1000 × A470 − 2.05 × Ca − 114.8 × Cb)/245

Total chlorophyll content was calculated as the sum of chlorophyll a and chlorophyll b. Pigment contents were expressed as mg g^−1^ FW and calculated as C × V/(1000 × m), where C is the pigment concentration in the extract (μg mL^−1^), V is the extraction volume (mL), and m is the tissue fresh mass (g).

### 4.5. Leaf Hormone Measurements

Leaves used for hormone analysis were rinsed and blotted dry using the same procedure described in Section 4.4. The samples were placed in a tissue grinder with grinding tubes until the tissue was completely broken into a homogeneous slurry. The homogenate was then centrifuged at 5000× *g* for 10 min at 2–8 °C, and the supernatant was collected. Immunoreactive estimates of gibberellin (GA), indole-3-acetic acid (IAA), cytokinin (CTK), and brassinosteroids (BR) were obtained using ELISA kits (Meike Biotechnology Co., Ltd., Shanghai, China; Meike, 96T format; catalog numbers: MK0035ZA for GA, MK0501OA for IAA, MK0510OA for CTK, and MK0444OA for BR). All four kits used competitive ELISA and targeted GA_3_, IAA, zeatin, and brassinolide, respectively, according to the manufacturer. The BR assay showed manufacturer-reported cross-reactivity of 4.37% with epibrassinolide. Four biological replicates were analyzed per treatment, with three technical replicates for each sample. Calibration standards supplied with each kit were assayed alongside the samples. Mean absorbance at 450 nm was calculated for each standard and sample and normalized to the zero-concentration standard as %B/B_0_ = 100 × A_450_/A_450,0_, where A_450,0_ is the mean absorbance of the zero-concentration standard. Sample concentrations were determined from the corresponding calibration curves according to the manufacturer’s instructions. Samples were diluted five-fold during the assay, and the calculated concentrations were corrected for this dilution. The kit-supplied calibration standards were 0, 7.5, 15, 30, 60, and 120 pmol L^−1^ for GA; 0, 2.5, 5, 10, 20, and 40 nmol L^−1^ for IAA; 0, 3, 6, 12, 24, and 48 ng mL^−1^ for CTK; and 0, 15, 30, 60, 120, and 240 pg mL^−1^ for BR. The manufacturer-reported assay sensitivities were 1 pmol L^−1^, 0.1 nmol L^−1^, 0.1 ng mL^−1^, and 1 pg mL^−1^, respectively. Hormone immunoreactive estimates were normalized to tissue fresh mass using the equation H = C × D × V/m, where H is the immunoreactive estimate per gram fresh weight, C is the concentration obtained from the calibration curve, D is the dilution factor, V is the extraction volume, and m is the tissue fresh mass. The volume unit used for V was matched to the concentration unit used for C. Immunoreactive estimates were expressed as pmol g^−1^ FW for GA, nmol g^−1^ FW for IAA, ng g^−1^ FW for CTK, and pg g^−1^ FW for BR. Spike-and-recovery tests, dilution-linearity assessments, and matrix-effect validation were not performed for *N. thermarum* leaf extracts. Therefore, all four hormone measurements are reported as immunoreactive estimates rather than chemically verified endogenous hormone concentrations. Cross-reactivity of the BR assay with the applied EBL was not experimentally assessed; consequently, the BR estimate cannot distinguish endogenous BR from any contribution of applied EBL or other cross-reacting compounds.

### 4.6. Transcriptome Sequencing and Analysis

After 3 weeks of treatment, leaf blade and petiole tissues were collected from plants in the BR400 (400 nM) treatment group and the control (CK) group, immediately frozen in liquid nitrogen, and stored at −80 °C for later use. Each sample group contained four biological replicates, totaling 16 samples. Total RNA was extracted using the TRIzol method. RNA quality and concentration were assessed by agarose gel electrophoresis and a NanoDrop 2000 spectrophotometer(Thermo Fisher Scientific, Waltham, MA, USA), and then sequencing was performed by Beijing Novogene Technology Co., Ltd. (Beijing, China) on an Illumina NovaSeq 6000 platform (Illumina, Inc., San Diego, CA, USA) with paired-end 150 bp reads.

Raw reads were processed using fastp v0.19.7 with the parameters “-g-q 5-u 50-n 15-l 150” for adapter and poly-G trimming and quality, ambiguous-base, and length filtering. Clean reads were aligned to the *N. thermarum* reference genome (GCA_011799765.1) using HISAT2 v2.0.5. Gene-level quantification was performed using featureCounts v1.5.0-p3. According to the project completion reports, reads with mapping quality below 10, reads not aligned as pairs, and reads mapping to multiple genomic locations were excluded. FPKM values were used to summarize expression abundance, whereas raw gene counts were used for differential expression analysis. Differential expression analysis was performed separately for leaves and petioles using raw counts and DESeq2 v1.20.0, with BR400 compared with CK within each tissue. *p*-values were adjusted using the Benjamini–Hochberg method. Genes with adjusted *p* ≤ 0.05 and |log_2_ fold change| ≥ 1 were classified as differentially expressed genes (DEGs). Shared DEGs were identified by intersecting the original gene identifiers from the two tissue-specific DEG sets, and their directions of change were compared using the corresponding log_2_ fold changes. Complete DEG lists and shared DEG statistics are provided in Supplementary Tables S1–S3. GO and KEGG enrichment analyses were performed separately for the upregulated and downregulated DEG sets using NovoMagic with clusterProfiler v3.8.1. Enrichment was evaluated using hypergeometric tests, with *p*-values adjusted using the Benjamini–Hochberg method and adjusted *p* < 0.05 defining significant enrichment. The background comprised genes included in differential expression analysis that had annotations in the corresponding database. GeneRatio was defined as k/n, where k is the number of input DEGs associated with a term or pathway and n is the effective annotated input size for the corresponding analysis. BgRatio was defined as M/N, where M is the number of background genes associated with that term or pathway, and N is the effective annotated background size. GO denominators were specific to the BP, CC, and MF categories. The corresponding input and background sizes are provided in Supplementary Table S18. The exported KEGG results linked *N. thermarum* gene identifiers to Arabidopsis thaliana identifiers and used Arabidopsis-specific pathway codes with the “ath” prefix. Within each enrichment record, contributing *N. thermarum* identifiers were distinct. Different *N. thermarum* genes mapping to the same Arabidopsis identifier remained separately represented in the exported counts; no additional collapsing was performed during preparation of the supplementary tables. These annotations were treated as putative functional assignments rather than evidence of one-to-one orthology or experimentally validated functional equivalence. Complete enrichment outputs for all, upregulated, and downregulated DEG sets are provided in Supplementary Tables S4–S15. Principal component analysis (PCA) was performed separately for each tissue using raw counts exported from NovoMagic. Genes with fewer than 10 total counts across the eight samples within a tissue were excluded. Counts were variance-stabilized using PyDESeq2 v0.5.4 with use_design = False. PCA was calculated using the 500 genes with the highest variance after transformation, with gene centering and without scaling to unit variance. All biological replicates were retained. For Figure 9, tissue-specific log_2_ fold changes in selected shared DEGs were rounded to two decimal places for display. Heatmap color saturation was capped at ±6 without altering the underlying values.

RT-qPCR was performed using the same RNA samples used for RNA-seq, comprising four biological replicates for each treatment (CK and BR400) within each tissue (leaf blade and petiole). One microgram of total RNA was reverse-transcribed using the HiScript II 1st Strand cDNA Synthesis Kit (Vazyme Biotech Co., Ltd. Nanjing, Jiangsu, China, R212-02) according to the manufacturer’s instructions. RT-qPCR was performed using ChamQ Universal SYBR qPCR Master Mix (Vazyme Biotech Co., Ltd. Nanjing, Jiangsu, China, Q711-02) in a 20 μL reaction volume. Expression was normalized to *NtActin7*, and relative expression was calculated using the 2^−ΔΔCt^ method, with CK within each tissue as the calibrator. Primer sequences are provided in Supplementary Table S19.

### 4.7. Statistical Analysis

Morphological, pigment, and hormone immunoreactive estimate data were analyzed using SPSS 22.0 (IBM, Armonk, NY, USA). Technical replicate measurements were averaged within each biological replicate before analysis. Each treatment comprised four biological replicates, with one plant representing one biological replicate. Treatment effects were evaluated using one-way ANOVA, followed by the Waller–Duncan multiple range test with a Type I/Type II error seriousness ratio (K-ratio) of 100. Data are presented as means ± SD. Different lowercase letters indicate differences detected by the Waller–Duncan test. Residual normality and variance homogeneity were assessed using Shapiro–Wilk and mean-centered Levene’s tests (α = 0.05). Sensitivity analyses used Welch’s ANOVA with Games–Howell comparisons for leaf area, chlorophyll b, and carotenoids, and wild-bootstrap Welch tests for longitudinal leaf radius (200,000 resamples; max-t adjustment across all 15 treatment pairs). Adjusted *p* < 0.05 defined significant pairwise differences in the sensitivity analyses. One-way ANOVA results, Waller–Duncan groupings, assumption-test results, and sensitivity analyses are provided in Supplementary Table S16.

## 5. Conclusions

Exogenous 24-epibrassinolide promoted vegetative growth in *N. thermarum*, with 400 nM producing the highest mean longitudinal leaf radius, petiole length, and leaf area among the concentrations tested. Hormone immunoreactive estimates showed treatment-dependent patterns, with the highest mean GA and BR estimates occurring under BR400, but neither differed significantly from CK; the highest mean IAA estimate occurred under BR600. Transcriptome analysis identified 1053 DEGs in leaves and 2028 in petioles, including 512 shared DEGs. Enrichment analyses revealed tissue- and direction-dependent changes in cell wall-related gene expression, enrichment of phenylpropanoid biosynthesis among upregulated DEGs, and enrichment of photosynthesis–antenna proteins among downregulated DEGs in both tissues. Under BR400, leaf chlorophyll a and total chlorophyll concentrations were lower than CK, although the implications for photosynthetic performance remain unresolved. These findings establish the responsiveness of vegetative organ growth to EBL in *N. thermarum* and identify candidate genes and processes for further investigation. Functional, anatomical, and physiological studies are needed to determine how these responses contribute to organ growth and to assess their value for ornamental cultivation.

## Data Availability

Supplementary data supporting this study are provided in the Supplementary Materials. The raw RNA-seq reads from all 16 samples have been submitted to the NCBI Sequence Read Archive (SRA) under BioProject accession PRJNA1542475.

## Supplementary Materials

The following supporting information can be downloaded at: https://www.mdpi.com/article/10.3390/plants15203205/s1. Figure S1. RT-qPCR analysis of selected genes in *Nymphaea thermarum* leaves and petioles under BR400 relative to CK. Table S1. Differentially expressed genes in leaves under BR400 relative to CK. Table S2. Differentially expressed genes in petioles under BR400 relative to CK. Table S3. Shared differentially expressed genes between leaves and petioles. Table S4. GO enrichment results for all differentially expressed genes in leaves. Table S5. GO enrichment results for upregulated differentially expressed genes in leaves. Table S6. GO enrichment results for downregulated differentially expressed genes in leaves. Table S7. KEGG enrichment results for all differentially expressed genes in leaves. Table S8. KEGG enrichment results for upregulated differentially expressed genes in leaves. Table S9. KEGG enrichment results for downregulated differentially expressed genes in leaves. Table S10. GO enrichment results for all differentially expressed genes in petioles. Table S11. GO enrichment results for upregulated differentially expressed genes in petioles. Table S12. GO enrichment results for downregulated differentially expressed genes in petioles. Table S13. KEGG enrichment results for all differentially expressed genes in petioles. Table S14. KEGG enrichment results for upregulated differentially expressed genes in petioles. Table S15. KEGG enrichment results for downregulated differentially expressed genes in petioles. Table S16. One-way ANOVA results, Waller–Duncan groupings, assumption checks, and sensitivity analyses. Table S17. Per-library RNA-seq quality-control and alignment statistics. Table S18. Annotated input and background sizes for GO and KEGG enrichment analyses. Table S19. Primer sequences used for RT-qPCR.
