# Supplementary material for: Exogenous Brassinosteroid Promotes Vegetative Growth in the World’s Smallest Water Lily (Nymphaea thermarum)
Source: Plants (Basel). 2026 Oct 19;15(20):3205. doi: 10.3390/plants15203205 (PMC13709161; doi:10.3390/plants15203205)
Supplement: Supplementary file 1 [file plants-15-03205-s001.zip › Supplementary_Fig_S1.pdf]

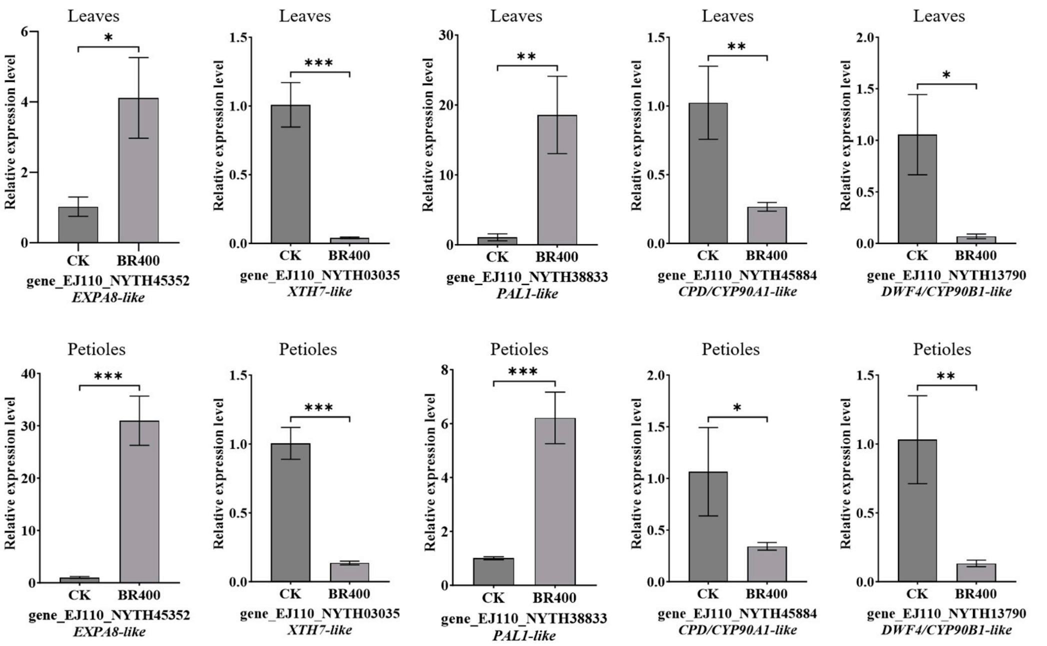

Figure S1. RT-qPCR analysis of selected genes in Nymphaea thermarum leaves and petioles under BR400 relative to CK. The genes were EXPA8-like (EJ110_NYTH45352), XTH7-like (EJ110_NYTH03035), PAL1-like (EJ110_NYTH38833), CPD/CYP90A1-like (EJ110_NYTH45884), and DWF4/CYP90B1-like (EJ110_NYTH13790). Upper and lower panels show leaves and petioles, respectively. Relative expression was normalized to *NtActin7* using the $2^{-\Delta\Delta Ct}$ method, with the corresponding CK group as the calibrator. Data are means ± SD from four biological replicates. *P < 0.05, **P < 0.01, and ***P < 0.001.
